# A Panel of Exosome-Derived miRNAs of Cerebrospinal Fluid for the Diagnosis of Moyamoya Disease

**DOI:** 10.3389/fnins.2020.548278

**Published:** 2020-09-25

**Authors:** Gang Wang, Yunyu Wen, Oluwasijibomi Damola Faleti, Qingshun Zhao, Jingping Liu, Guozhong Zhang, Mingzhou Li, Songtao Qi, Wenfeng Feng, Xiaoming Lyu

**Affiliations:** ^1^Department of Neurosurgery, Nanfang Hospital, Southern Medical University, Guangzhou, China; ^2^Department of Laboratory Medicine, The Third Affiliated Hospital, Southern Medical University, Guangzhou, China

**Keywords:** moyamoya disease, cerebrospinal fluid, exosomal miRNAs, biomarker, diagnosis

## Abstract

**Background:**

Moyamoya disease (MMD) is an important cause of stroke in children and young adults in Asia. To date, diagnosis remains challenging due to varying clinical manifestations and unknown pathogenesis. The study aims to identify cerebrospinal fluid (CSF) exosomal microRNAs (exomiRs) that can serve as a novel diagnostic biomarker for diagnosis and assess its clinical applications.

**Methods:**

CSF samples were taken from 31 MMD patients and 31 healthy controls. Initial screening of miRNA expression was performed on samples pooled from MMD patients and controls using microarray and validated using quantitative reverse transcription polymerase chain reaction (qRT-PCR). The diagnostic accuracy of the potential exosomal miRNAs was evaluated using receiver operating characteristic curve analyses in an independent patient cohort. The potential pathways regulated by the miRNAs was also determined using bioinformatics analysis.

**Results:**

The microarray results demonstrated that six exomiRs were dysregulated in the MMD patients compared to the controls. Using qRT-PCR, we validated four of the miRNAs (miR-3679-5p, miR-6165, miR-6760-5p, and miR-574-5p) as a biomarker for MMD diagnosis. The four exomiRs showed enhanced sensitivity (75%) and specificity (93.75%) in terms of differentiating MMD patients from healthy subjects [area under the curve (AUC) = 0.9453]. Pathway enrichment analysis for potential targets of six exomiRs identified proteins involved in cell adhesion and junction formation in the brain.

**Conclusions:**

We identified a novel and highly sensitive exomiRs signature for MMD detection and explored its potential targets using bioinformatics analysis.

## Introduction

Moyamoya disease (MMD) is an important cause of stroke in children and young adults in East Asian countries such as Korea, Japan, and China (Kim et al., 2016). An epidemiological survey of MMD in Japan found that the annual incidence of MMD is (0.35–0.94)/100,000, and the prevalence rate is (3.2–10.5)/100,000 (Baba et al., 2008). However, the pathogenesis of MMD is not clear. It is characterized by the stenosis of the internal carotid arteries which results in the formation of hazy vascular networks (moyamoya vessels) at the base of the brain (Bang et al., 2016). The patients showed different clinical manifestations which can be attributed to variation in affected to variation in patient age, the degree, and progression of stenosis and region of the cerebral cortex affected. In adult patients, intracranial hemorrhage as well as cerebral ischemia, cognitive dysfunction, epilepsy, involuntary movement, and headache are frequently observed (Kuroda and Houkin, 2008; Scott and Smith, 2009). One of the treatment options is revascularization surgery which is aimed at improving blood flow (Karasawa et al., 1992; Miyamoto et al., 1998). However, adult patients are often vulnerable to postoperative complication (Miyamoto et al., 2014; Ishii et al., 2018).

Exosomes are lipid-bilayer enclosed extracellular vesicles (30–100 nm in size) which act as a mediator of intercellular communication through the delivery of proteins and microRNAs (exomiRs) (Colombo et al., 2014). Exosomes are released by most cells of the body and are capable of modulating important central nervous system (CNS) processes including neuronal development, maintenance, and regeneration (Chivet et al., 2013). In recent years, the role of exosomes under neuropathologies has received significant attention (Candelario and Steindler, 2014; Jan et al., 2017). Studies have shown that exosomes are involved in neuroinflammation and disruption of the blood–brain barrier (BBB) (Gupta and Pulliam, 2014; Xu et al., 2017). They also participate in the transfer of the toxic proteins and dysregulated miRNAs between resident brain cells and peripheral cells (Gui et al., 2015; Chen et al., 2017). Furthermore, exosomal miRNAs are protected from RNase degradation and can be detected at minute quantities in the cerebrospinal fluid (CSF). Given the importance of the exosomes in brain communication and under pathophysiological conditions, exosomal miRNAs (exomiRs) have emerged as an essential diagnostic tool.

As a consequence of the unclear complex manifestations, identification of biomarkers for diagnosis and a better understanding of MMD’s etiology has emerged as a vital area of research (Bersano et al., 2016). Accurate and early diagnosis of MMD is critical because the neurological status at diagnosis affect long-term outcome. Currently, diagnosis of MMD depends on neuroimaging techniques, such as Digital Subtraction Angiography (DSA), which provides information about the structure and physiology of the brain; however, it is invasive (Rice, 1994; Fukui, 1997). There is still an unmet need for a clinical biomarker to improve diagnosis and disease management (Kang et al., 2010; Sung et al., 2018). A reliable biomarker with a high degree of sensitivity and specificity can offer a complementary and cost-effective means for MMD diagnosis, prognosis, and counseling.

To date, there has not been any report about the diagnostic potential of exosomal miRNAs (exomiRs) in CSF for diagnosis of MMD. Based upon this paradigm, we profiled and uncovered four differentially regulated CSF exosomal miRNAs in MMD patients. Furthermore, we examined its diagnostic potential in an independent patient cohort and the potential function of the miRNAs using bioinformatic analysis. The study shows that four CSF exomiRs that can complement currently in-use neuroimaging techniques for MMD diagnosis and also provides insight into the role of CSF exomiRs in MMD pathology.

## Materials and Methods

### Patients and Samples Collection

From October 2017 to September 2018, 31 patients (20 males and 11 females) were admitted to Nanfang Hospital with MMD. 31 patients diagnosed with MMD by DSA were selected as the experimental group in Nanfang Hospital. The diagnostic criteria for MMD were based on the guidelines published in 2012 by the Research Committee on the Spontaneous Occlusion of the Circle of Willis of the Ministry of Health and Welfare, Japan. The clinical characteristics of 31 MMD patients are similar. Another 31 patients with no obvious intracranial disease were selected as the control group. The study was carried out using CSF collected from MMD patients who needed bypass surgery and control patients. The 31 control patients are patients who need spinal anesthesia and suffer from either fracture of lower limbs, varicose veins in lower extremity or knee arthroplasties. These patients were meet the following criteria: (1) No obvious intracranial disease diagnosed by CT; (2) No CNS related history. CSF were obtained before injecting anesthetic via lumbar puncture. The CSF was placed in a 15 ml RNase/DNase free centrifuge tube. The whole CSF was centrifuged at 1,500 × *g* for 10 min at room temperature, and the supernatant was transferred to a 15 ml RNase/DNase free centrifuge tube. The specimens were then stored in −80°C. The study was approved by the Review Committee of the Nanfang Hospital Ethics Committee of Southern Medical University (NFEC-201906-K11), and informed consent was obtained from the patients.

### ExoQuick Extraction of Cerebrospinal Fluid Exosomes

We isolated exosomes from the CSF of all patients by using ExoQuick precipitation (System Biosciences Inc., Mountain View, CA, United States) following the manufacturer’s instructions. In brief, the exosomes were pelleted by adding the exosome extraction reagent and centrifuging at 1,500 × *g* for 10 min at 4°C. The exosomal-particles were resuspended in 10 mM PBS in four times the volume of CSF. Dynamic light scattering (DLS) analysis and transmission electron microscopy (TEM) were used to identify exosomes.

### Western Blot Analysis

Exosomes were lysed in RIPA Buffer (Sigma, R0278). Protein concentrations were determined using a BCA Protein Assay Kit (Solarbio Life Science, PC0020; Beijing, China). Protein extracts were separated by 10% sodium dodecyl sulfate-polyacrylamide gel electrophoresis (SDS-PAGE) at 80 V for 2 h and blotted onto a polyvinylidene di-fluoride (PVDF) membrane (Millipore, IPVH00010; Billerica, MA, United States) for 100 min at 320 mA. The membranes were then blocked for 60 min with 5% BSA (Solarbio Life Sciences, A8020) in 0.1% Tween-20 (Sigma, P9416) in TBS. Subsequently, the membranes were incubated with primary antibodies at 4°C overnight, followed by incubation with horseradish peroxidase (HRP)-labeled secondary antibodies (Cell Signaling Technology, Danvers, MA, United States) at room temperature for 1 h. The immunoreactive bands were visualized using Immobilon ECL Ultra Western HRP Substrate (Millipore, WBULS0500) and imaged using the Tanon-5500 Chemiluminescent Imaging System (Tanon Science & Technology; Shanghai, China). The anti-CD63(ab217345), anti-Calnexin (ab227310), anti-TSG101 (ab125011), and anti-Alix (ab186429) were obtained from Abcam (Cambridge, MA, United States).

### miRNA Extraction From Exosomes

Total RNA was extracted from the CSF exosomes and purified using mirVana^TM^ miRNA Isolation Kit without phenol (Ambion, Austin, TX, United States) following the manufacturer’s instructions. The RNA concentration was quantified using a NanoDrop spectrophotometer (Thermo Fisher Scientific, Waltham, MA, United States). The integrity and quality of RNA were assessed by an Agilent Bioanalyzer 2100 (Agilent Technologies, Santa Clara, CA, United States), and the RNA samples with an RNA integrity number ≥6.0 and 28S/18S >0.7 were deemed acceptable to perform the miRNA microarray assay and reverse transcription.

## Microarray Analysis

Exosomes extracted from equal amounts of CSF from two sources (15 relatively healthy patients, 15 MMD patients before revascularisation surgery) were pooled into five individual samples per group and used for miRNA microarray analysis. The microarray hybridization, data generation, and normalization were performed by Shanghai Biochip Corp. According to the standard Agilent protocols. Human miRNA microarrays from Agilent Technologies, which contain probes of 1,887 human miRNAs from the Sanger database v.18.0, were used in this study. Visualization of microarray data was performed using MeV 4.6 software (MultiExperiment Viewer)^1^. A miRNA was considered overexpressed if the expression in pooled MMD cases samples was >1.5-fold higher than that in pooled controls. The overexpressed miRNAs were considered candidate miRNA biomarkers for further analysis. The microarray data are available in Gene Expression Omnibus with the accession number: GSE129792.

### Quantitative Reverse Transcription Polymerase Chain Reaction (qRT-PCR) for Validation

q-PCR was performed using Mir-X^TM^ miRNA quantitative reverse transcription polymerase chain reaction (qRT-PCR) TB Green^TM^ Kit (Takara, Japan). Primers used in this study were as follows: hsa-miR-574-5p: forward primer 5′-TGAGTGTGTGTGT GTGAGTGTGT-3′; hsa-miR-3679-5p: forward primer 5′-TGAGGATATGGCAGGGAAGGGG A-3′; hsa-miR-6124: forward primer 5′-GGGAAAAGGAAGGGGG AGGA-3′; hsa-miR-6165: forward primer 5′-CAGCAGGAG GTGAGGGGAG-3′; hsa-miR-6760-5p: forward primer 5′-CAGGGAGAAGGTGGAAGTGCAGA-3′. The reverse primers are using the reagents provided by the kit Mir-X^TM^ miRNA qRT-PCR TB Green^TM^ Kit (Takara, Japan).

### ROC Analysis for Differential Expression of Circulating miRNAs

To assess the potential diagnostic value of identified miRNAs, a receiver operating characteristic (ROC) curve analysis was performed. First, we plot the ROC curve for an individual factor, and then we calculated the probability by performing binary logistic analysis on two of the significantly different miRNAs and use the ROC curve analysis for the newly generated probability value. Similarly, ROC curve analyses were performed for three of the four miRNAs.

### miRNAs Downstream Target Analysis

Predict the downstream genes of the above four miRNAs on four prediction websites: TargetScan, miRDB, miRTarbase, Tarbase. Summarize all downstream genes predicted by the above website. Then, difference integration analysis (Venn analysis) was performed.

### Statistical Analysis

Results are presented as means ± SEM, and the statistical analysis was performed between the sham control and the MMD groups using Mann–Whitney test. *P*<0.05 was counted as significant.

## Results

### Characteristics of the Study Population

In total, 31 MMD patients and 31 non-MMD donors were enrolled in the present study. The baseline characteristics of the MMD patient group and healthy control group in the screening and validation sets are listed in Table 1. There were no significant differences in the distribution of most routine blood biochemical parameters, such as AST (*P* = 0.992) and HDL (*P* = 0.647) between the two groups in either the screening or validation sets. However, significant differences were observed in the RBC (*P* = 0.011) and Hb (*P* = 0.01) levels.

**TABLE 1:** Clinical characteristics of study population (31 controls and 31 MMD cases).

	Non-MMD	MMD	*P*-value
Male sex (microarray)	8(53.3%)	9(60.0%)	0.983
Male sex (validation)	8(50.0%)	11(68.8%)	0.529
Age	41.8 ± 17.7	38.7 ± 14.2	0.581
WBC (×10^9^)	8.2 ± 3.0	7.7 ± 1.7	0.641
RBC (×10^9^)	4.4 ± 0.7	4.9 ± 0.5	0.011
Hb (mmol/L)	126.0 ± 19.5	143.4 ± 15.5	0.01
Glu (mmol/L)	5.0 ± 0.9	4.9 ± 0.6	0.338
K^+^ (mmol/L)	4.1 ± 0.4	4.0 ± 0.4	0.25
Na^+^ (mmol/L)	140.2 ± 2.0	141.0 ± 2.4	0.315
ALT (U/L)	19.3 ± 10.2	35.2 ± 26.2	0.031
AST (U/L)	25.0 ± 19.5	24.9 ± 11.8	0.992
TB (umol/L)	10.9 ± 5.5	9.1 ± 3.9	0.289
DB (umol/L)	4.5 ± 2.1	3.8 ± 1.7	0.3
TC (mg/DL)	160.0 ± 33.7	185.5 ± 29.0	0.365
TG (mg/DL)	88.7 ± 21.3	214.5 ± 50.2	0.16
HDL (mg/DL)	46.4 ± 10.9	42.5 ± 5.0	0.647
LDL (mg/DL)	99.4 ± 25.7	114.5 ± 19.1	0.475

*WBC, white blood cell; RBC, red blood cell; Hb, hemoglobin; Glu, glucose; LDL, low density lipoprotein; HDL, high density lipoprotein; TB, total bilirubin; DB, direct bilirubin; TC, total cholesterol; TG, triglyceride.*

### Isolation and Identification of Exosomes Derived From CSF

**FIGURE 1:**
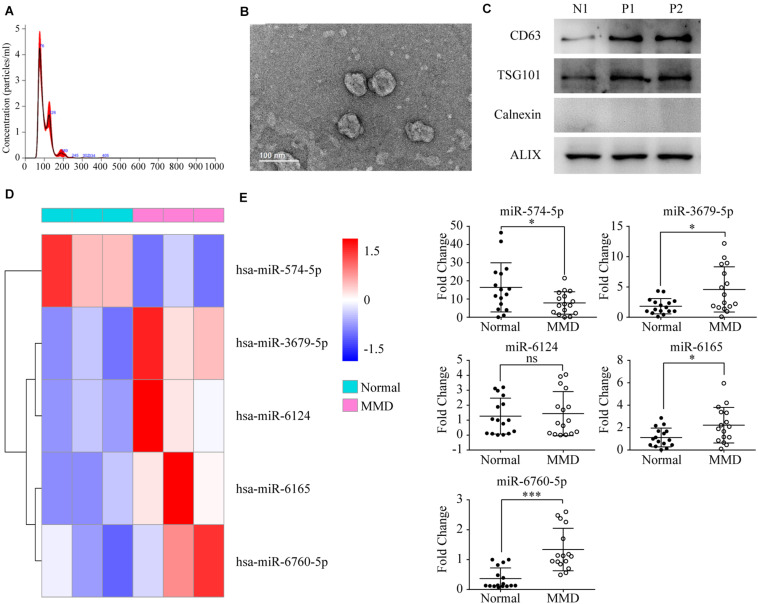
Characterization of CSF-Exos in MMD patients: **(A)** Particle size distribution measured by DLS. **(B)** Morphology observed by TEM. Scale bar: 100 nm. **(C)** Western blotting and quantitative analysis of the exosome surface markers (N1: Normal 1, P1: Patient 1, P2: Patients 2). **(D)** Heat map of microRNA (miRNA) microarray expression data from CSF samples of individuals with (*n* = 15) and without (*n* = 15) Moyamoya Disease (MMD). miRNA expression is hierarchically clustered on the *y* axis, and CSF samples from individuals with and without MMD are hierarchically clustered on the *x* axis. The legend on the right indicates the miRNA represented in the corresponding row. The relative miRNA expression is depicted according to the color scale shown on the right. Red indicates up-regulation; and blue, down-regulation. **(E)** Validation of microRNA (miRNA) microarray data by quantitative reverse-transcription polymerase chain reaction. The microarray cohort included 16 individuals with MMD and 16 controls without Moyamoya Disease (non-MMD). The *P*-values were calculated by Mann–Whitney test. (*: *P* < 0.05; ***: *P* < 0.001; ns: non-sense).

Dynamic light scattering, TEM, and western blotting were used to characterize the particles secreted into CSF. The results showed that most of these vesicles ranged from 50 to 120 nm in size (Figure 1A). In TEM experiments with CSF-Exos, the results showed that the vast majority of these nanoparticles exhibited a cup- or sphere-shaped morphology (Figure 1B), indicating the presence of exosomes. These hollow spherical microvesicles were further confirmed by western blotting. The expression of the exosome markers CD63, TSG101 and Alix (Figure 1C) were significantly enriched in exosomes. All these data indicate that exosomes were successfully isolated.

### Selection of Differential microRNAs Using Microarray

To identify a diagnostic miRNA signature for MMD, we performed miRNA microarray analysis using exosomal microRNAs extracted from the CSF samples of 15 MMD patients and 15 healthy subjects. Due to the limited sample availability and the low level of exosomal miRNAs in CSF, we pooled three patients samples into one group. A total of five groups of samples was used for the analysis. The microarray contained 1,887 miRNAs probes, and a miRNA was considered overexpressed if there is a 1.5-fold difference in its expression in pooled MMD cases samples compared to controls. After applying the filtering criteria, we identified four up-regulated and one down-regulated microRNAs (hierarchical clustering analysis seen in Figure 1D). In sum, the results suggest that miRNAs were differentially expressed in MMD patients compared to healthy controls. We selected five miRNAs (miR-574-5p, miR-3679-5p, miR-6124, miR-6165, miR-6760-5p) for further validation by quantitative RT-PCR in an independent patient cohort.

### Validation of Target microRNAs/Validation of Candidate microRNAs by qRT-PCR

To confirm the microarray results, we performed PCR validation using 16 MMD and 16 healthy control CSF samples. The clinical characteristics of the patients are summarized in Table 1. There were no significant differences in biochemical parameters between the two groups. microRNAs (miR-574-5p, miR-3679-5p, miR-6124, miR-6165, miR-6760-5p) which showed average signal value equal or greater 1.5, were used for the qRT-PCR assay validation. We found that all but one of the five microRNAs showed the same expression patterns as shown in microarray analysis. miR-3679-5p, miR-6165, miR-6760-5p were up-regulated, and miR-574-5p, was down-regulated (Figure 1E). In total, these data confirm the validity of differentially expressed exosomal miRNAs in CSF and suggest that the identified miRNAs may play a functional role in the pathogenesis of MMD.

### Diagnostic Utility of Potential microRNAs

**FIGURE 2:**
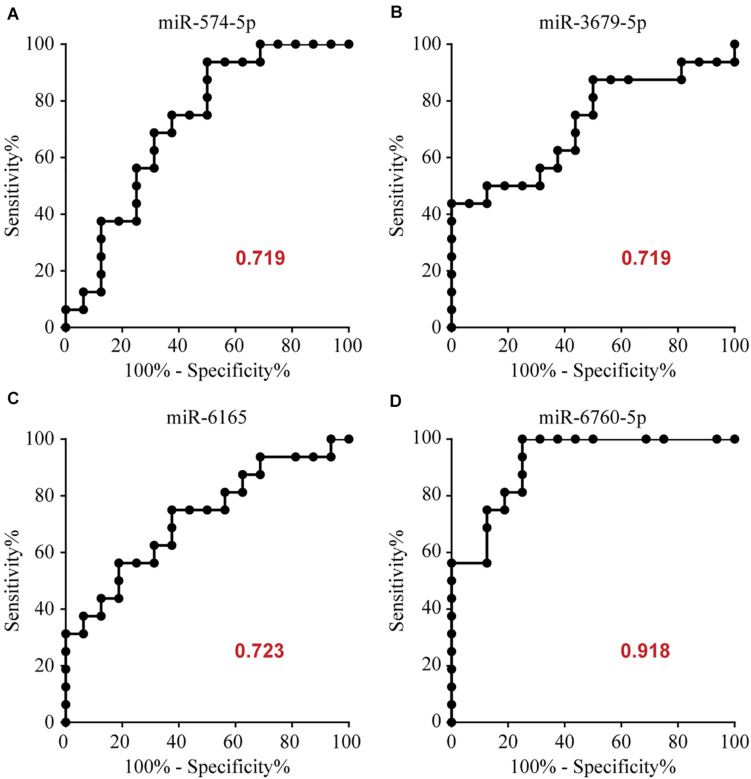
Area under the curve (AUC) analysis of receiver-operating characteristics. The AUC (values given on the graphs) for miRNAs with significantly different circulating levels was calculated for the MMD group.

**FIGURE 3:**
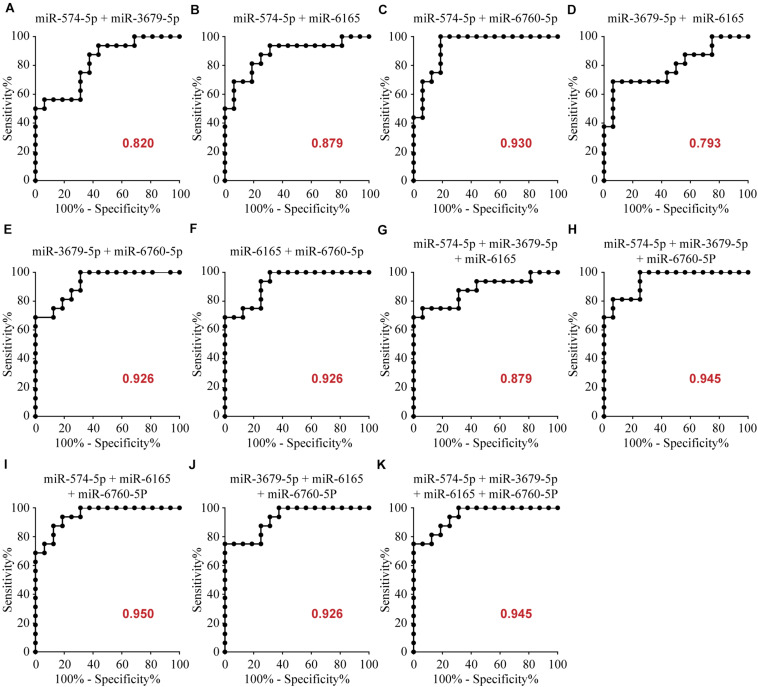
Area under the curve (AUC) analysis of combination of these miRNAs. The AUC (values given on the graphs) for combination of these miRNAs with significantly increased circulating levels was calculated for the MMD group.

To determine the diagnostic values of the significantly different expression miRNAs (miR-574-5p, miR-3679-5p, miR-6165, miR-6760-5p), we plotted ROC curves and calculated the area under the curve (AUC). The details of the AUC, sensitivity, and specificity of each miRNA marker can be found in Supplementary Table S1. miR-574-5p, miR-3679-5p, miR-6165, miR-6760-5p had significant diagnostic power for differentiating MMD patients from healthy controls. Their AUC values were 0.719, 0.719, 0.723, 0.918, respectively (Supplementary Table S1 and Figure 2). To further explore the applicability of the exosomal miRNAs as potential diagnostic biomarkers of MMD, we combined the four miRNAs. These combinations improved the diagnostic performance compared with individual miRNA or pair combinations (Figure 3 and Supplementary Table S2). We then selected the combination of miR-574-5p, miR-6165, miR-6760-5p, which had better diagnostic power than other combinations (Figure 3 and Supplementary Table S2). The diagnostic accuracy of the combined three miRNAs (miR-574-5p, miR-6165, miR-6760-5p) was an AUC of 0.9492, a sensitivity of 87.5% and a specificity of 87.5%.

### Bioinformatics Analysis for Selected miRNAs and Their Targets

**FIGURE 4:**
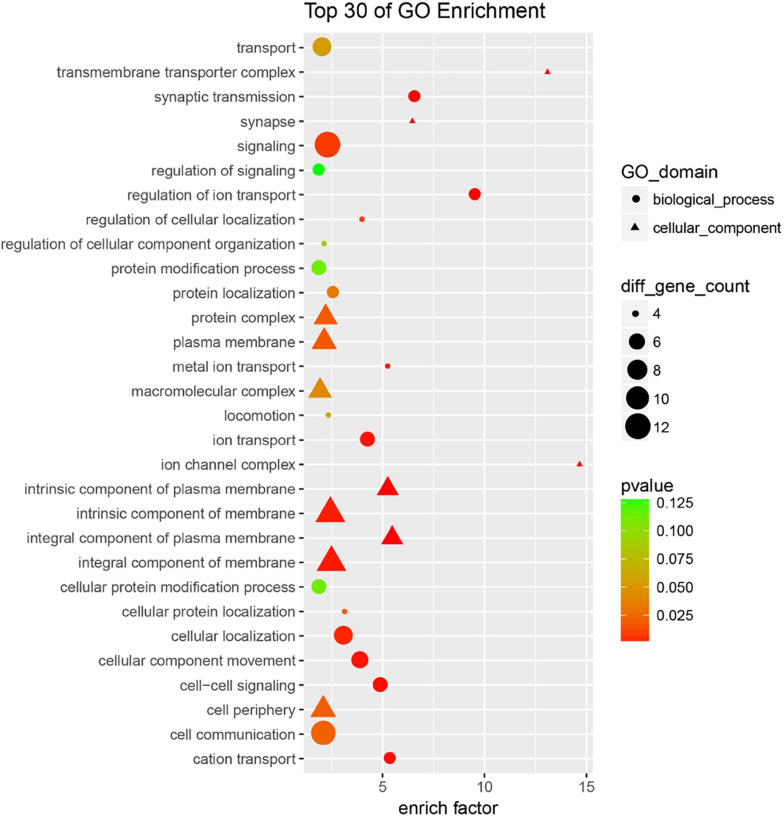
GO analysis of overlapping targets of these candidate miRNAs. The overlapping targets were used for GO analysis, top 30 functional annotations were show up according to the enrich factor and *P*-value.

To further understand the potential biological functions of four miRNAs, we identified the overlapping targets using GO analysis and KEGG pathway enrichment analyses. The results of GO classification indicate that most of the common target genes were involved in cell membrane-related components, signal transduction, and ion transport (and its regulation) (Figure 4 and Supplementary Table S3). Also, pathway enrichment analysis (Supplementary Table S4) showed that the most significant dysfunctional pathways were in cell adhesion. Other significant targets are related to tight junctions, adherens junction (AJ), axon guidance, and circadian rhythm. The results indicated that the four miRNA classifiers might participate in the MMD through the regulation of the series of pathways in the brain.

### miRNAs Downstream Target Analysis

**FIGURE 5:**
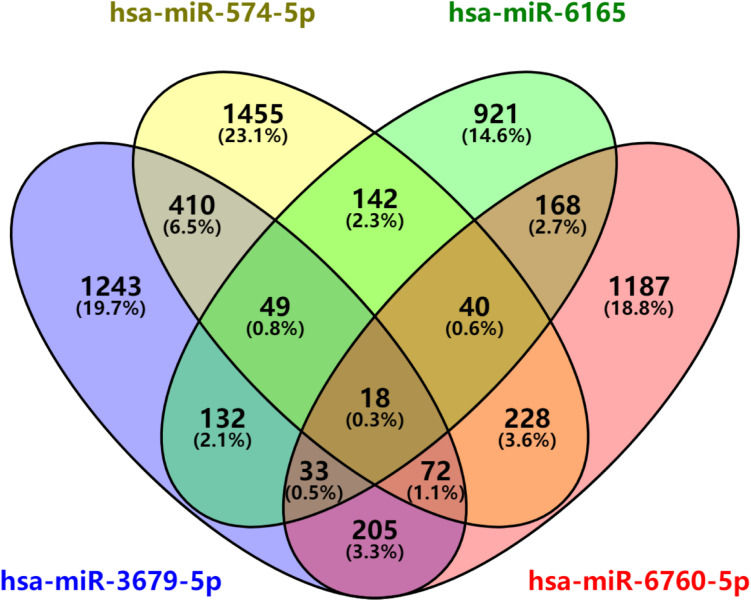
miRNAs downstream target analysis. Venn diagram analyses of four independent databases reveals four possible targets of miR-3679-5p, miR-6165, miR-574-5p, and miR-6760-5p.

After predicted in four websites, we found that there are 2162 downstream target genes for hsa-miR-3679-5p, 2414 downstream target genes for hsa-miR-574-5p, 1503 downstream target genes for hsa-miR-6165 and 1951 downstream target genes for hsa-miR-6760-5p (Supplementary Table S5). VENN analysis revealed that 18 mRNAs can be used as the downstream target genes of the above four miRNAs: ASTN2, CD84, CNTN2, CRY2, CTDNEP1, IYD, KCNE1, KIF1B, MLEC, MPP2, PLXNA2, PVRL1, RAB3B, RSPO4, SCN4B, SHISA6, TEAD1, ZDHHC9 (Figure 5).

## Discussion

Exosomes have emerged as an essential vehicle for cell-to-cell communication between the neurons and other nervous system cells including astrocytes and neurovascular endothelial cells (Budnik et al., 2016). It is a promising source of biomarkers for early diagnosis and management of pathophysiological conditions including cancer, neurodegenerative diseases, and infection. Dai et al. (2014) detected the expression of miRNA in serum of patients with MMD. However, Yagi et al. (2017) found that some miRNAs can only be detected in CSF, and suggesting that miRNAs in this fraction might be derived from brain tissue, their study provides an essential baseline for miRNA biomarker studies of neurological diseases. The reasons why we choose CSF instead of serum are as follows: Firstly, MMD is not only a vascular disease, but also a CNS disease. Therefore, CSF is the biologic fluid in closest contact with the CNS (Wildenauer et al., 1991; Romeo et al., 2005). Secondly, blood is related to multiple systems and organs throughout the body, diseases of other systems may affect the composition of biomarker in serum. Therefore, we hold that the detection of miRNA in the CSF of patients can better represent the pathological state of MMD. Previous studies have reported the altered expression of specific miRNAs in the CSF exosomes of patients affected by neurodegenerative diseases (Kocerha et al., 2014; Reed et al., 2018). Nonetheless, the role of exosomes in the pathogenesis of MMD is unknown. To ascertain whether CSF exosomal miRNA expression signature can distinguish MMD patients from healthy controls, we conducted miRNA expression profiling using microarray followed by validation using qRT-PCR assay (Reed et al., 2018).

We identified, for the first time, four miRNAs with altered expression in exosomes derived from CSF of MMD patients: miR-574-5p, miR-3679-5p, miR-6165, miR-6760-5p. We assessed the diagnostic potential of the miRNAs in an independent set of patients samples with MMD (*n* = 16) and healthy controls (*n* = 16) using a logistic regression model. The combination of three miRNAs (miR-574-5p, miR-6165, miR-6760-5p), showed a good clinical performance (AUC = 0.9492) with a sensitivity of 87.5% and a specificity of 87.5% for predicting MMD. The dysregulated miRNAs have previously been implicated in cases of neuroinflammation and deteriorated synaptic function, subarachnoid hemorrhage, acute stroke and hematoma enlargement (Zheng et al., 2012; Hu et al., 2018). Of note, three of the four dysregulated miRNAs are up-regulated and thus suggest there is a repression of the targets in the enriched pathways.

Understanding the pathogenesis of MMD and identification of clinical biomarkers are two crucial areas of MMD research. To identify the putative miRNA-target genes and pathways, we performed bioinformatics analysis. The predicted targets for the miRNA were found to be two cell adhesion molecules (CAMs); namely nectin 1 and contactin 2. Nectin 1 is an immunoglobulin-like transmembrane CAM expressed by many cell types including neurons. It binds with itself and other CAMs at cell-cell interfaces through homophilic and heterophilic interactions, respectively (Sakisaka et al., 2007). Nectin 1 plays a vital role in the formation of AJs which in turn support the development of tight junctions through the recruitment of adhesion molecules (Indra et al., 2013). The assembly and architecture of TJs are dependent on the formation and maintenance of AJs (Dejana, 2004). Tight junctions are involved in the movement of ions and solutes across BBB paracellular spaces, and its disruption has been linked to BBB dysfunction (Coisne and Engelhardt, 2011). Nectin-1 is also involved in the formation of a synapse between neurons and axonal guidance during development (Mandai et al., 2015).

Contactin-2/TAG-1 is a neural adhesion molecule that belongs to the immunoglobulin superfamily (Zuko et al., 2011). It is a glycan phosphatidylinositol (GPI)-anchored protein which contains N-terminal Ig-like domains and fibronectin type III (FNIII) repeats. The Ig-like and fibronectin domains serve as building blocks for extracellular proteins involved in cell adhesion (Compton et al., 2008). Contactin-2 is expressed in the juxtaparanodal region of myelinated axons, oligodendrocytes, and Schwann cells (Traka et al., 2002). They support the assembly and architecture of juxtaparanodal junctions of the nodes of Ranvier in myelinated nerves and the K+ channels (Stogmann et al., 2013). Its expression has also been observed in neurons found in the gray matter of the hippocampus, entorhinal cortex, cerebellum, and olfactory bulb and the spinal cord (Soares et al., 2005). Contactin-2 has been implicated in neurodegenerative diseases, such as Alzheimer’s disease (AD), multiple sclerosis, and loss of cognitive abilities in contactin-2 knockout mice (Derfuss et al., 2009).

As previously reported, transient neurologic symptoms (TNS) is observed in 17–61% of MMD patients after direct bypass surgery. The observation has been linked to unstable BBB, vasogenic edema and local cortical hyperperfusion (Hamano et al., 2017). Excessive degradation of the vascular matrix by MMPs (Indra et al., 2013), a high level of VEGF has been linked to the unstable BBB observed in moyamoya patients (Sakamoto et al., 2008). Literature suggests that nectin-1 plays are involved in the formation of tight junction in BBB (Coisne and Engelhardt, 2011). However, further investigation is needed to clarify the role of the dysregulated miRNAs and their target proteins in the MMD pathogenesis.

There are several limitations to our study. MMD incidence shows some association with the geographical location. However, the study only examined Chinese MMD patients. Also, we pooled the CSF samples of the patients for the initial microarray screening because of the limited amounts of samples, cost, and low concentration of the exosomes in the CSF. Although sample pooling has its limitation which includes the inability to account for biological variation within individual patient samples, it has employed in numerous biological experiments, and its efficacy has been statistically investigated (Cheng et al., 2013). Of note, to cater for the limitation imposed by this method, we assessed the each of miRNAs in single samples using PCR and only miRNAs which are differentially regulated in the same pattern in both pooled and individual samples were selected for further analysis. More importantly, we identified a unique four exosomal miRNA signature which showed good diagnostic accuracy in clinical validation. This work will serve as the basis for clinical studies with a larger patient cohort to demonstrate the accuracy of the 5-biomarker panel as a promising diagnostic test for MMD.

## Conclusion

In summary, our study which conducted on MMD patients from Asia (China) showed for the first time that CSF exosomal miRNAs have the potential to complement current MMD diagnostic tools and improve MMD care. Aside from the exosomal miRNA signature, we also provide readers of Frontiers in Neuroscience insight about the possible roles of nectin-1 and contactin-2 in MMD pathogenesis.

## Data Availability Statement

The datasets presented in this study can be found in online repositories. The names of the repository/repositories and accession number(s) can be found in the article/Supplementary Material.

## Ethics Statement

The ethical standards of the Review Committee of the Nanfang Hospital Southern Medical University. Written informed consent to participate in this study was provided by the participants’ legal guardian/next of kin. Written informed consent was obtained from the individual(s), and minor(s)’ legal guardian/next of kin, for the publication of any potentially identifiable images or data included in this article.

## Author Contributions

GW, YW, and OF conducted most of the bench work, assembled the results, and wrote the manuscript. QZ, JL, GZ, ML, and SQ performed clinical specimens. YW performed total RNA extraction and qRT-PCR. WF and XL provided funds and ideas. All authors contributed to the article and approved the submitted version.

## Conflict of Interest

The authors declare that the research was conducted in the absence of any commercial or financial relationships that could be construed as a potential conflict of interest.

## Footnotes

1 http://www.tm4.org/mev/
