# Supplementary material for: A Panel of Exosome-Derived miRNAs of Cerebrospinal Fluid for the Diagnosis of Moyamoya Disease
Source: Front Neurosci. 2020 Sep 25;14:548278. doi: 10.3389/fnins.2020.548278 (PMC7546773; doi:10.3389/fnins.2020.548278)
Supplement: Supplementary file 1 [file Data_Sheet_1.ZIP › Supplementary files/Supplementary Table 2.pptx]

## Slide 1
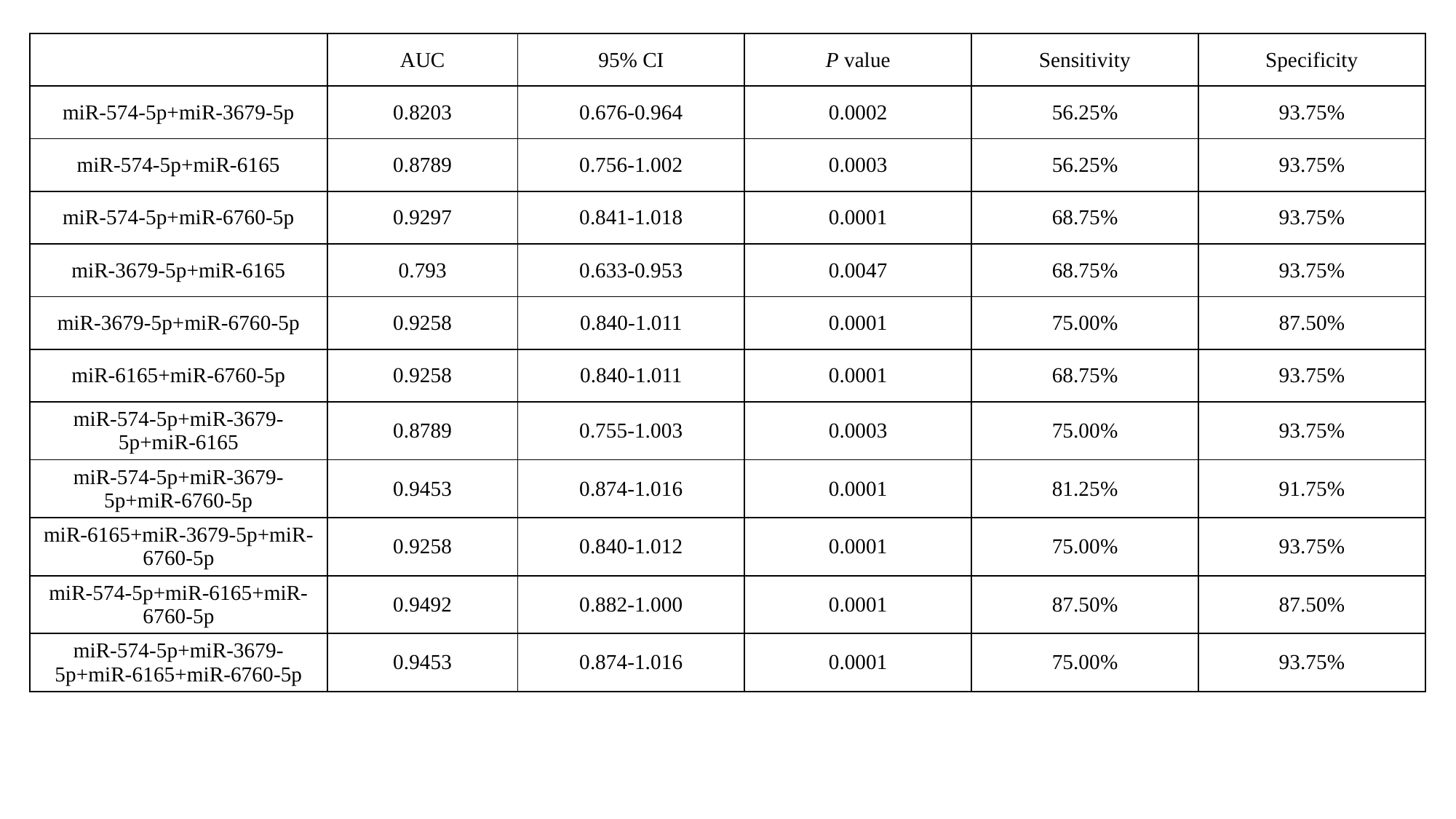

| | AUC | 95% CI | P value | Sensitivity | Specificity |
| --- | --- | --- | --- | --- | --- |
| miR-574-5p+miR-3679-5p | 0.8203 | 0.676-0.964 | 0.0002 | 56.25% | 93.75% |
| miR-574-5p+miR-6165 | 0.8789 | 0.756-1.002 | 0.0003 | 56.25% | 93.75% |
| miR-574-5p+miR-6760-5p | 0.9297 | 0.841-1.018 | 0.0001 | 68.75% | 93.75% |
| miR-3679-5p+miR-6165 | 0.793 | 0.633-0.953 | 0.0047 | 68.75% | 93.75% |
| miR-3679-5p+miR-6760-5p | 0.9258 | 0.840-1.011 | 0.0001 | 75.00% | 87.50% |
| miR-6165+miR-6760-5p | 0.9258 | 0.840-1.011 | 0.0001 | 68.75% | 93.75% |
| miR-574-5p+miR-3679-5p+miR-6165 | 0.8789 | 0.755-1.003 | 0.0003 | 75.00% | 93.75% |
| miR-574-5p+miR-3679-5p+miR-6760-5p | 0.9453 | 0.874-1.016 | 0.0001 | 81.25% | 91.75% |
| miR-6165+miR-3679-5p+miR-6760-5p | 0.9258 | 0.840-1.012 | 0.0001 | 75.00% | 93.75% |
| miR-574-5p+miR-6165+miR-6760-5p | 0.9492 | 0.882-1.000 | 0.0001 | 87.50% | 87.50% |
| miR-574-5p+miR-3679-5p+miR-6165+miR-6760-5p | 0.9453 | 0.874-1.016 | 0.0001 | 75.00% | 93.75% |
