# Supplementary figures and images for: A Panel of Exosome-Derived miRNAs of Cerebrospinal Fluid for the Diagnosis of Moyamoya Disease
Source: Front Neurosci. 2020 Sep 25;14:548278. doi: 10.3389/fnins.2020.548278 (PMC7546773; doi:10.3389/fnins.2020.548278)

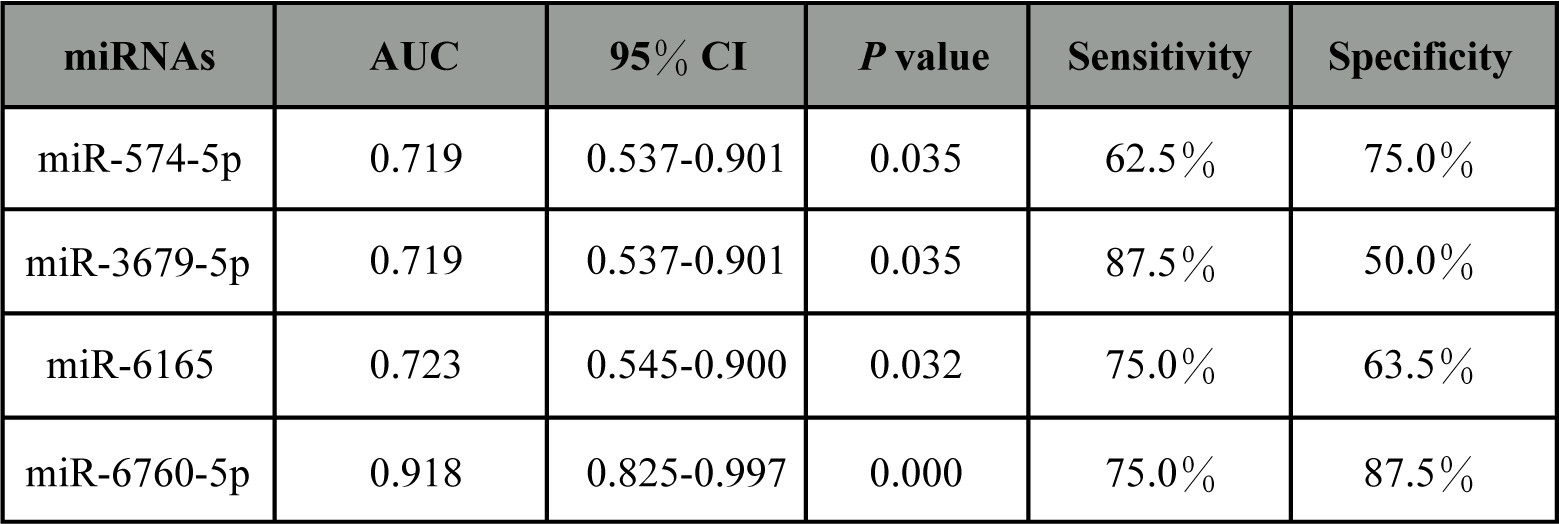

Supplement: Supplementary file 1 [file Data_Sheet_1.ZIP › Supplementary files/Supplementary Table 1.tif]
