# Supplementary material for: A Panel of Exosome-Derived miRNAs of Cerebrospinal Fluid for the Diagnosis of Moyamoya Disease
Source: Front Neurosci. 2020 Sep 25;14:548278. doi: 10.3389/fnins.2020.548278 (PMC7546773; doi:10.3389/fnins.2020.548278)
Supplement: Supplementary file 1 [file Data_Sheet_1.ZIP › Supplementary files/legends.doc]

**Legends：**

**Supplementary Table 1. Sensitivity and Specificity of the regulated miRNAs in the CSF of MMD and non-MMD individuals.** Data were evaluated via a cut-off point.

**Supplementary Table 2. Sensitivity and specificity of the combined miRNAs in the CSF of MMD and non-MMD individuals.** Data were evaluated via a cut-off point.

**Supplementary Table 3. GO analysis of overlapping targets of these candidate miRNAs.**

**Supplementary Table 4. KEGG pathway enrichment analyses of overlapping targets of these candidate miRNAs.**

**Supplementary Table 5. miRNAs downstream target analysis**
